# Examining the factor structure of the Physical Literacy for Life self-assessment tool (PL4L) among Japanese adults and its relationship with the stages of change model for participation in regular physical activity

**DOI:** 10.3389/fpubh.2025.1505502

**Published:** 2025-03-12

**Authors:** Misaki Matsunaga, Koya Suzuki, Masahiro Matsui, Kenta Toyama, Shizuo Ito, Yoshinori Okade, Kosho Kasuga, Pengyu Deng, Tetsuya Matsuo, Yasunori Morioka, Hiroshi Aono, Hisashi Naito

**Affiliations:** ^1^Graduate School of Health and Sports Science, Juntendo University, Chiba, Japan; ^2^Institute of Health and Sports Science & Medicine, Juntendo University, Chiba, Japan; ^3^Tokyo Marathon Foundation, Tokyo, Japan; ^4^Faculty of Sport Culture, Nippon Sport Science University, Tokyo, Japan; ^5^Faculty of Education, Gifu University, Gifu, Japan; ^6^College of Sport and Wellness, Rikkyo University, Saitama, Japan; ^7^College of Sports Sciences, Nihon University, Tokyo, Japan; ^8^Sport Sciences Laboratory, Japan Sport Association, Tokyo, Japan

**Keywords:** physical literacy, Physical Literacy for Life self-assessment tool, stages of the change model, factor structure, internal consistency, confirmatory factor analysis

## Abstract

**Introduction:**

Physical literacy contributes to physical activity and comprises four conceptually interrelated domains: physical, emotional, social, and cognitive. The International Sport and Culture Association proposed the Physical Literacy for Life self-assessment tool (PL4L); however, its factor structure and usability are not clear. This study aimed to examine the factor structure and internal consistency of the PL4L in adults and its association with the stages of change model for participation in regular physical activity.

**Methods:**

For this study, a total of 940 Japanese adults (age: 41.8 ± 13.2 years) completed a web-based cross-sectional survey. Physical literacy (PL) was assessed using the PL4L. The stages of change model regarding participation in regular physical activity were examined using a self-reported questionnaire that applied the Transtheoretical Model. Internal consistency was assessed by Cronbach’s α and McDonald’s ω. Factor structure was evaluated using confirmatory factor analysis. Structural equation modeling was used to investigate the relationship between the PL4L and the stages of change for participation in regular physical activity. Internal consistency coefficients were found to be high.

**Results:**

In the confirmatory factor analysis, the modified model, incorporating theoretically justified error covariances, demonstrated a good fit. The factor loadings between domains and items were all significant. Significant associations were also identified between PL and stages of change for participation in regular physical activity.

**Discussion:**

The PL4L’s factor structure is consistent with the concept of physical literacy among Japanese adults, which correlates with the stages of change for participation in regular physical activity. Future studies should investigate whether addressing PL can effectively increase physical activity levels.

## 1 Introduction

Physical literacy (PL) is a concept that contributes to the ability to perform physical activity (1). The International Physical Literacy Association defines PL as “the motivation, confidence, physical competence, knowledge, and understanding to value and take responsibility for engagement in physical activities for life” (2). It encompasses four conceptually interrelated domains, including physical (physical competence and skills, etc.), psychological or emotional (confidence, motivation, etc.), social (ethics, social skills, etc.), and cognitive (knowledge, strategy, etc.) (3–5). A previous study indicated that PL, being related to physical activity, may provide opportunities for health promotion and disease prevention (1). In school-age children, PL is positively associated with not only physical activity (6) but also health indicators such as body composition, fitness, blood pressure, and Health-Related Quality of Life (4, 7). A holistic exercise and education intervention that addressed the physical, behavioral, emotional, and cognitive components of physical activity was shown to improve PL and physical activity behavior among inactive adults (8), suggesting that PL acquisition enhances physical activity.

In Japan, the concept of PL has recently started gaining recognition. For the first time, the Third Basic Sports Plan, formulated in 2022 (9, 10), mentioned PL as a goal for fostering children’s lifelong engagement in sports and physical activity. Additionally, the Japan Sports Association (JSPO), organization responsible for promoting sports in Japan, has initiated a PL-related research project in 2021 (11). However, despite these advancements, the development and implementation of PL in Japan remain in their early stages compared to PL-leading countries such as Canada and Australia.

Previous studies have attempted to develop assessment tools for PL (12, 13). The first assessment tool for adults was used to assess the PL of physical education teachers (14). The United Nations Educational, Scientific, and Cultural Organization (UNESCO) promotes Quality Physical Education (QPE) (15). Previous studies in the field of education have suggested that QPE and PL are closely aligned, with the latter contributing positively to achieving QPE goals (16). It was thought that physical education teachers needed to understand the concept of PL for students to live active and healthy lives. Given that PL may promote physical activity, an assessment tool was also developed for use in young adults, such as university students. A previous study identified seven instruments for measuring PL in adults, three of which are original tools (13). Of these three, two target young adults, while one, the Perceived Physical Literacy Instrument (PPLI), was designed to assess physical education teachers (14). More recently, the Perceived Physical Literacy Questionnaire (PPLQ) has been validated as a PL assessment tool for adults in Germany (17). Currently, there is no validated PL assessment tool for Japanese adults; this highlights a need for future research to develop tools suitable for diverse populations and languages.

The International Sports and Culture Association (ISCA) proposed the Physical Literacy for Life self-assessment tool (PL4L) to enable the general European public to self-assess their PL levels. It is available on the ISCA website for public viewing, response, and feedback (18). The PL4L was developed based on the PL concept and consists of four domains (physical, emotional, cognitive, and social). Originally in English, it has been translated into multiple languages, including Slovenian, Spanish, Bulgarian, and French, and can be easily completed online. Although this tool is accessible and useful, to our knowledge, no published studies have examined its factor structure or internal consistency in any language. Furthermore, the potential correlation between its psychometric properties and physical activity has not yet been examined. Therefore, this study aimed to examine the factor structure and internal consistency of the PL4L in adults and its association with the stages of the change model for participation in regular physical activity.

The Transtheoretical Model represents the phases through which individuals progress when making conscious changes to their health behavior (19). These stages reflect variations in awareness and actions related to sustaining health behaviors. Therefore, by evaluating the relationship between the stage of change model for physical activity participation and PL, this study seeks to deepen the understanding of how PL impacts both the amount of physical activity and an individual’s awareness and readiness to engage in such behaviors. Thus, this study offers a valuable framework for assessing how PL influences habitual health behaviors.

## 2 Methods and measures

### 2.1 Participants

In this cross-sectional examination, young and middle-aged Japanese adults (aged 18–64 years) were recruited by an Internet panel company^1^ registering over 3.35 million non-volunteer panelists. The company updates its panel registration information every 6 months. For this study, it randomly selected 7,300 Japanese adults from its database, ensuring equal distribution of participants by age and sex (display rate 0.21%). Additionally, these panelists received an email requesting survey participation between November 1 and 6, 2023. Among them, 1,132 completed the survey (participation rate, 15.5%). After excluding 114 adults with missing or inconsistent data, 68 adults were further excluded by the company to ensure an equal distribution of 20 participants per age group and sex. Eventually, 940 adults (470 men, 470 women) were included. The present study was approved by the Juntendo University School of Health and Sports Science and the Graduate School of Health and Sports Science Research Ethics Committee (no. 2023–125). All participants consented to participate in the study and completed the questionnaire.

### 2.2 Demographic information

Age and sex data were provided by the internet panel company. Additional demographic information, including height, weight, and education level (≧ 16 years or < 16 years), was collected using self-reported questionnaires. The participants were asked to report their height in centimeters, and their weight in kilograms. Education level was determined by asking whether the participant had completed college education. Body mass index (BMI) was calculated by dividing weight (kg) by height squared (m^2^).

### 2.3 Physical literacy

Physical Literacy was assessed using the PL4L, developed in English by the ISCA (18). We were granted permission by the ISCA to create a Japanese version of the PL4L and commissioned an independent professional translator for its back-translation. The final version was confirmed by ISCA. The PL4L consists of 16 items: six related to the physical domain, four to the emotional domain, and three each to the cognitive and social domains. Each item offers choices ranging from Level 1 to Level 3, allowing participants to select the level that best suits them. The first question and its response options for each domain are outlined below (the complete set of questions and answer options is available in Supplementary file 1).

- Physical domain 1 (Strength)
  Capacity of muscle(s) to exert force against an object
  - Level 1 option: I have difficulty using my strength in simple daily activities (e.g., cannot carry shopping bags or do a sit up; cannot keep correct posture while seated).
  - Level 2 option: I can use my strength in general contexts of physical activity (e.g., push-ups, sit-ups, pull-ups, gardening/shoveling).
  - Level 3 option: I’m able to use my strength in challenging contexts of physical activity (e.g., lifting heavy weights, rock climbing, circuit training, vigorous activity).
- Emotional domain 1 (Motivation)
  Reasons for engaging in movement and physical activity
  - Level 1 option: I do not feel like participating in physical activities/movement.
  - Level 2 option: I participate in physical activity because it brings me approval, recognition or rewards from others.
  - Level 3 option: I participate in physical activity because it brings me joy, pleasure and self-realization.
- Cognitive domain 1 (Knowledge)
  Factual knowledge and information that a person knows and can convey about physical activities (e.g., knowing that benefits of physical activity include physical, emotional, social and cognitive benefits)
  - Level 1 option: I have difficulty recognizing benefits of physical activity.
  - Level 2 option: I know the general benefits of physical activity.
  - Level 3 option: I can relate different types of physical activities with their specific benefits (e.g., sports, rhythmic activities, active commuting).
- Social domain 1 (Ethic)
  Moral principles that govern a person’s behavior relating to fairness and justice
  - Level 1 option: I have difficulty recognizing principles of fairness, respect and inclusion in physical activities.
  - Level 2 option: I generally apply principles of fairness and inclusion in physical activities.
  - Level 3 option: I can use strategies to improve conditions for respect, fairness and inclusion in physical activities.

In this study, the questions were scored as follows: 1 point for Level 1 answers, 2 points for Level 2 answers, and 3 points for Level 3 answers. The scores for the four domains and the total score were calculated, with the physical domain score ranging from 6 to 18, the emotional domain score from 4 to 12, and the cognitive and social domain scores from 3 to 9. The total score ranged from 16 to 48.

### 2.4 Stages of change model for participation in regular physical activity

The stage of change model for participation in regular physical activity was determined using a self-report questionnaire (20, 21). Regular physical activity was defined as performing a physical activity such as walking and sports for over 20–30 min per session and more than 2–3 times per week (21). Participants selected one of the following five options: “I currently do not exercise and do not intend to exercise in the next 6 months” (pre-contemplation); “I currently do not exercise, but I intend to exercise within the next 6 months” (contemplation); “I currently get some exercise, but not regularly” (preparation); “I currently exercise regularly, but I have only begun doing so within the past 6 months” (action); and “I currently exercise regularly and have been doing so for longer than 6 months” (maintenance).

### 2.5 Data analysis

The original PL4L model assumes a four-factor structure. Therefore, confirmatory factor analysis with maximum likelihood estimation was conducted to investigate whether the data obtained in Japan conformed to the structure assumed by the original. The analysis modeled the four domains as latent variables and the corresponding items as observed variables. Several indices were used to assess the fit between the model and the data: goodness of fit index (GFI), adjusted goodness of fit index (AGFI), comparative fit index (CFI), and root mean square error of approximation (RMSEA). These indices were collectively evaluated to determine overall fit. GFI, AGFI, and CFI range from 0 (indicating poor fit) to 1 (indicating good fit), while RMSEA is considered acceptable at levels ≦ 0.08, and levels ≦ 0.05 are regarded as a very good fit (22). For a relative comparison among multiple models, the Akaike Information Criterion (AIC) was used (23), with smaller values indicating better-fitting models. The chi-square statistic is typically regarded as a measure of the badness of fit of models (24). However, due to the recommendation that alternative fit indices should be collectively considered, particularly in cases with large sample sizes, it was not used to assess model fit (25). To refine the model, we consulted the modification index, which estimates the expected decrease in the chi-square value after adjustments (22). Error covariances were introduced only when theoretically justifiable (22, 26), and the fit of the revised model was evaluated (22, 26). Factor loadings were considered to have substantial elements of common factors if their absolute values were greater than 0.40 (27). Additionally, a stratified analysis was conducted separately by sex subgroups. Confirmatory factor analyses were performed for each subgroup to assess model fit, using the same fit indices as in the overall analysis (GFI, AGFI, CFI, and RMSEA). Factor loadings were also examined, with values greater than 0.40 considered substantial.

Internal consistency for the total scale and its four domains was assessed using Cronbach’s α and McDonald’s ω. The coefficients (0 ≤ α, ω ≤ 1) were used to assess whether the PL4L items addressed the same concept, with a value of 0.7 or higher considered acceptable (28, 29).

Structural equation modeling was used to investigate the relationship between the Japanese version of PL4L and the stages of change for participation in regular physical activity. Each question item, serving as an observed variable, was assigned to one of the four domains and treated as a latent variable. A higher-order latent variable for PL was then assumed, and its relationship with the observed variable of the stages of change for participation in regular physical activity was analyzed. The model fit was assessed using the same indices as in the CFA (GFI, AGFI, CFI, and RMSEA), with maximum likelihood estimation. All coefficients reported in our analyses were standardized.

All statistical analyses in this study utilized data from the full sample of 940 participants. Analyses were performed using SPSS Statistics version 28 and SPSS AMOS version 26 (IBM Japan, Tokyo, Japan). The statistical significance level was set at less than 5%.

## 3 Results

The participant characteristics are presented in Table 1. The averages of age and BMI were 41.8 ± 13.2 years and 22.1 ± 3.90 kg/m^2^. The proportion of participants with ≧ 16 years of education was 54.5%. In the stages of change model for participation in regular physical activity, the action stage recorded the lowest rate (5.6%) and the maintenance stage recorded the highest rate (26.5%). Table 2 depicts the scores for each item and the total PL scores. The mean for each PL item ranges from 2.0 to 2.3. The scores for each domain were as follows: physical domain 12.8 ± 2.1, emotional domain 8.5 ± 1.9, cognitive domain 6.1 ± 1.5, and social domain 6.1 ± 1.4; the total score was 33.5 ± 5.7.

**Table 1:** Participants characteristics.

			Mean ± SD
Number of participants		n	940
Sex			
Men		n [%]	470 [50.0]
Women		n [%]	470 [50.0]
Age			
18–19		n [%]	40 [4.3]
20–29		n [%]	200 [21.3]
30–39		n [%]	200 [21.3]
40–49		n [%]	200 [21.3]
50–59		n [%]	200 [21.3]
60–64		n [%]	100 [10.6]
Education level			
Over 16 years		n [%]	512 [54.5]
Less than 16 years		n [%]	428 [45.5]
Height		cm	164.7 ± 8.83
Weight		kg	60.2 ± 13.25
BMI		kg/m^2^	22.1 ± 3.90
The stages of change model for participation in PA			
Pre-contemplation		n [%]	238 [25.3]
Contemplation		n [%]	210 [22.3]
Preparation		n [%]	190 [20.2]
Action		n [%]	53 [5.6]
Maintenance		n [%]	249 [26.5]

**Table 2:** Physical literacy mean and standard deviation.

		Mean ± SD
Physical 1	Strength	2.1 ± 0.39
Physical 2	Stamina	2.2 ± 0.49
Physical 3	Movement skills	2.2 ± 0.43
Physical 4	Movement using an object	2.1 ± 0.48
Physical 5	Coordination and balance	2.1 ± 0.39
Physical 6	Object manipulation skills	2.1 ± 0.40
Emotional 1	Motivation	2.3 ± 0.83
Emotional 2	Confidence	2.0 ± 0.50
Emotional 3	Physical regulation	2.1 ± 0.50
Emotional 4	Emotional regulation	2.1 ± 0.56
Cognitive 1	Knowledge	2.1 ± 0.55
Cognitive 2	Rules (and tactics)	2.1 ± 0.55
Cognitive 3	Strategy	2.0 ± 0.65
Social 1	Ethics	2.0 ± 0.52
Social 2	Society and culture	2.1 ± 0.50
Social 3	Collaboration	2.0 ± 0.53
Physical domain score	range: 6–18	12.8 ± 2.07
Emotional domain score	range: 4–12	8.5 ± 1.88
Cognitive domain score	range: 3–9	6.1 ± 1.52
Social domain score	range: 3–9	6.1 ± 1.37
Total score	range: 16–48	33.5 ± 5.74

**Figure 1:**
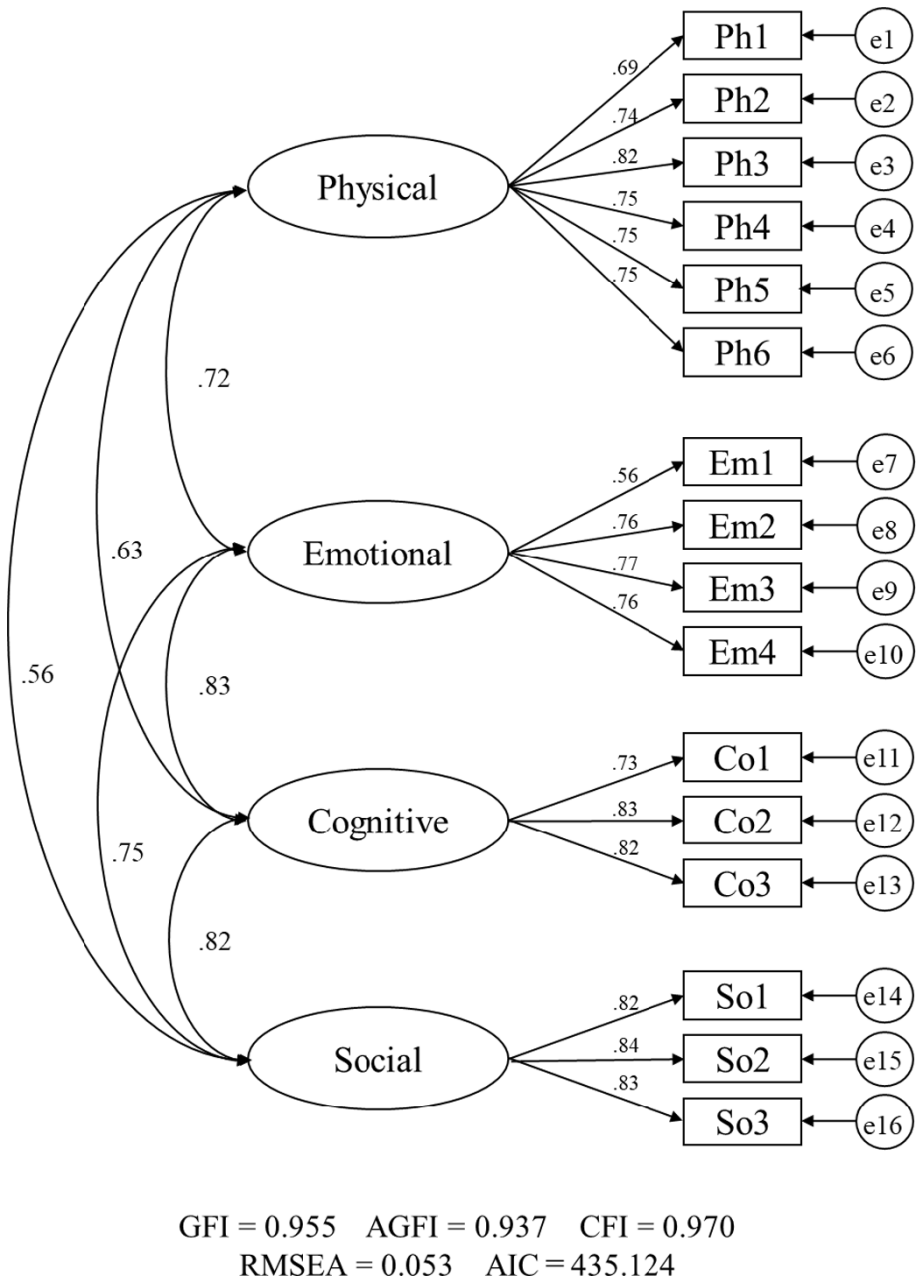
Final factor model of the Physical Literacy for Life self-assessment tool among Japanese adults.Goodness-of-fit indices (GFI, AGFI, CFI) suggest better model fit with higher values, whereas RMSEA indicates better fit with lower values. The Akaike Information Criterion (AIC), which is used to compare models, is lower for this model (AIC = 520.045) than for the initial model (AIC = 570.045), indicating an improvement. Factor loadings from latent variables to observed variables are meaningful when approximately 0.40 or greater, and all paths are statistically significant (*p* < 0.001).

In the confirmatory factor analysis, the structure of the PL4L was validated to conform to the assumed structure of the original version. The fit between the initial model and the data was as follows: GFI = 0.936, AGFI = 0.911, CFI = 0.955, RMSEA = 0.066, and AIC = 570.045. The modification indices were examined to guide the search for a model with a better fit. Although each fit index showed acceptable values, the modification indices between the error terms of Physical 5 (Coordination and Balance) and Physical 6 (Object Manipulation Skills) showed a potential for improving the model. The model was adjusted by assuming error covariance between these two items. Figure 1 depicts the modified factor model of the PL4L in Japanese adults. It provided an improved fit to the data: GFI = 0.955, AGFI = 0.937, CFI = 0.970, RMSEA = 0.053, AIC = 435.124. The factor loadings between domains and items ranged from 0.56 to 0.85, all of which were significant (*p* < 0.001) (27). In the stratified analysis, the model demonstrated acceptable fit in both, men and women subgroups. The fit indices for the men model were GFI = 0.937, AGFI = 0.912, CFI = 0.970, and RMSEA = 0.058, while those for the women model were GFI = 0.944, AGFI = 0.921, CFI = 0.962, and RMSEA = 0.053. Factor loadings ranged from 0.56 to 0.86 in both subgroups, with no items falling below 0.40. The CFA figures for each model are provided as Supplementary file 2.

The internal consistency coefficients are presented in Table 3. Cronbach’s α coefficient for all 16 items was 0.92. Analysis by domain revealed high internal consistency for the physical (α = 0.89), cognitive (α = 0.83), and social (α = 0.89) domains, and acceptable internal consistency for the emotional domain (α = 0.77). Similarly, McDonald’s ω coefficients also demonstrated good internal consistency, with values of 0.92 for all items, 0.89, 0.84, and 0.87 for the physical, cognitive, and social domains, respectively, while the emotional domain showed acceptable internal consistency (ω = 0.76).

**Table 3:** Internal consistency coefficient.

	Items	Cronbach’s α	McDonald’s ω
All Questions	16	0.92	0.92
Physical domain	6	0.89	0.89
Emotional domain	4	0.77	0.76
Cognitive domain	3	0.83	0.84
Social domain	3	0.89	0.87

**Figure 2:**
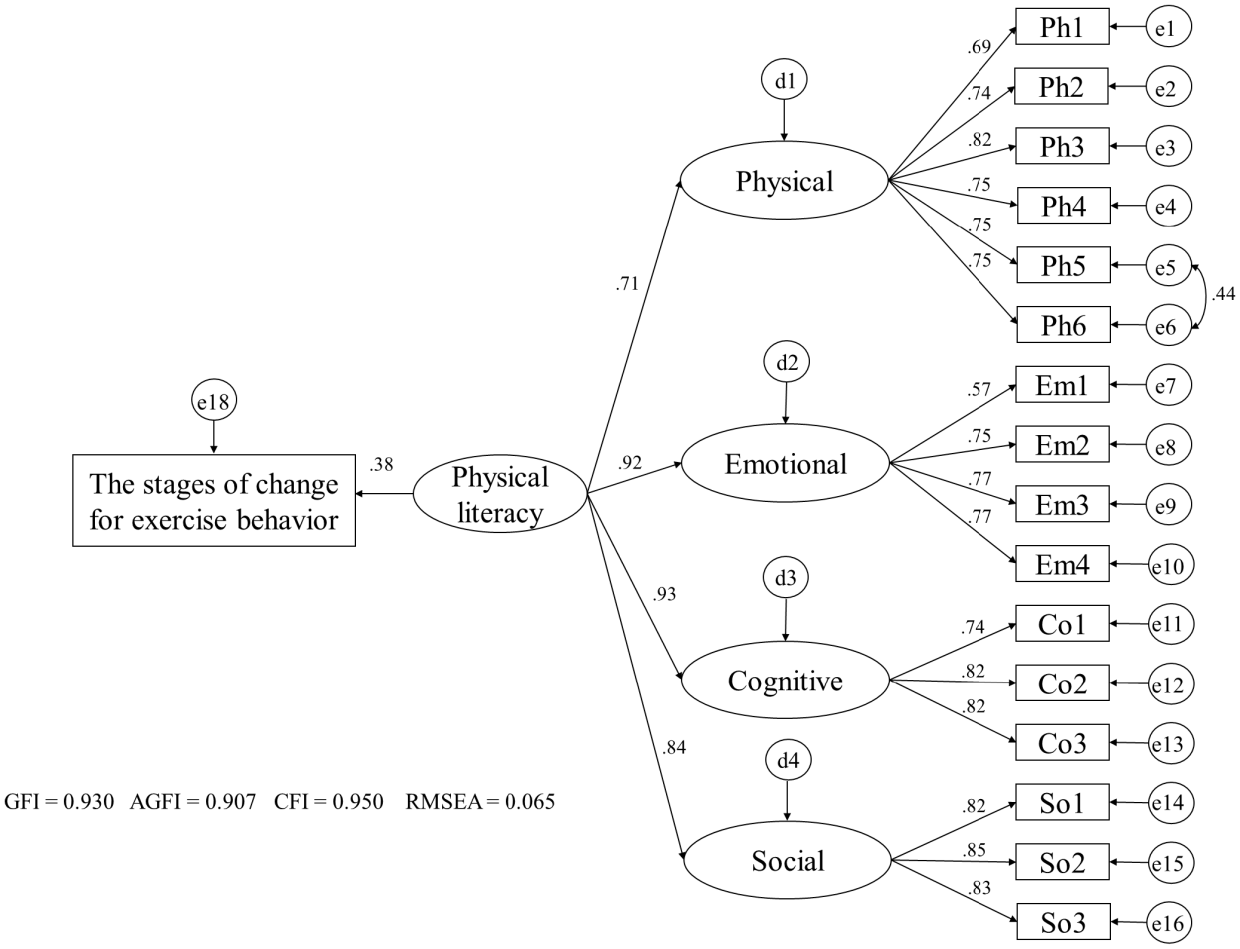
Influence of physical literacy on the stages of change for participation in physical activity.Goodness-of-fit indices (GFI, AGFI, CFI) suggest better model fit with higher values, whereas RMSEA indicates better fit with lower values. All paths are statistically significant (p < 0.001). The path coefficient from the hypothesized higher-order latent variable PL to the stages of change for participation in regular physical activity is 0.38.

Figure 2 depicts the relationship between PL and the stages of change for participation in regular physical activity. This study identified significant associations between PL and stages of change for participation in regular physical activity, with a coefficient of 0.38. The rest of the goodness-of-fit indices were acceptable (GFI = 0.930, AGFI = 0.907, CFI = 0.950, RMSEA = 0.065).

## 4 Discussion

This study examined the factor structure and internal consistency of the PL4L. Although the initial model revealed an acceptable fit in the CFA, we examined a revised model by considering the error covariance between the error variables of Physical 5 and 6 based on the modification indices. This examination led to a model with an improved fit. The error covariance can only be assumed when it can be interpreted practically (22, 26). In the PL4L, Physical 5 assesses coordination and balance, while Physical 6 evaluates object manipulation skills. Both items reflect the deft movements of the body, which is likely why the assumption of error covariance resulted in a better model fit. Moreover, in the preset model, the path coefficients between each domain and the observed variables were satisfactory overall (27). Based on these results, the PL4L, conforms to the theoretical model presented in the ISCA (18) and previous studies (3–5) and is considered factorially valid. Internal consistency analyses revealed that Cronbach’s α and McDonald’s ω of all items and each domain exceeded the threshold of 0.7, indicating satisfactory internal consistency (28, 29), indicating that the PL4L demonstrates a certain level of reliability for assessing young and middle-aged adults.

Most PL assessment tools have been developed in school settings, primarily targeting children and young adults (30). The development of adult PL assessment tools faced issues due to a disproportionate focus on certain countries (13) and a lack of established tools that cover a wide age range. The PL4L has been translated into multiple languages and is freely accessible online. Although many individuals use the PL4L to self-assess their PL, the scientific basis for its use as an assessment tool, including its psychometric properties such as factor structure and internal consistency, has not yet been published or made publicly available. Importantly, this study provided valuable insights into the factor structure and internal consistency of the PL4L in Japanese adults. It elucidates the tool’s practical utility for users and promotes its dissemination and adoption within the academic community. However, the PL questionnaire may need to be adapted to account for environmental and cultural contexts (31). For example, the Physical Literacy Assessment for Youth-Self (PLAY-Self) for Canadian youth includes a question about whether the respondent is good at sports and activities on ice and snow (32). Some countries receive snowfall in only some areas; in such cases, the level of PL is influenced by the area in which the respondent lives. The PL4L was deemed suitable for responding to questions, regardless of the specific environmental or cultural context. However, future research needs to examine whether PL assessed by the PL4L differs between countries.

Physical activity is an important health promoting behavior (33–35). However, existing strategies to promote regular physical activity are insufficient to change people’s behavior (36), highlighting the need for more innovative approaches. A previous review identified 12 barriers to and 18 facilitators of physical activity among university students (37). These include factors that are part of the four domains of PL that we aim to capture: (1) having the physical skills and fitness to participate in physical activity; (2) having the motivation, enjoyment, and self-efficacy to participate in physical activity; (3) knowledge about the benefits and ways of performing physical activity; and (4) exercising with others. Therefore, PL may serve as a gateway to physical activity, potentially impacting health. Currently, studies have found that interest in PL has moved beyond its traditional roots in physical education and extends to the field of public health (1, 38). This study revealed a positive relationship between PL and the stages of change model for participation in regular physical activity among adults. This result is consistent with that of a prior report on children, indicating that PL is associated with physical activity (6). Our findings suggest that PL is a new determinant of daily physical activity.

This study had several limitations. First, Internet panel surveys have limited generalization potential (39). To address this, the participants were recruited to ensure an equal distribution of age and sex. However, expectations related to incentives may influence the response quality. Additionally, this study employed a convenience sample, which does not fully represent the general population of Japanese adults. It would therefore be beneficial to conduct surveys using random sampling or complete enumeration in future studies. This study examined the factor structure and internal consistency of the PL4L in adults. However, the tool’s test–retest reliability, which evaluates its temporal stability (40), has not yet been confirmed. Future studies should address this limitation and report on the tool’s test–retest reliability. Additionally, this study employed self-report questionnaires, which may introduce social desirability and recall biases (41). It would be beneficial to conduct surveys using random sampling or complete enumeration in future studies. While this study assessed the stages of change model for physical activity based on self-reported data, it is important to note its limitations. Objective measures, such as wearable devices, may provide more accurate estimates of physical activity. These tools could offer a more comprehensive understanding of the role of PL in promoting sustained physical activity. Finally, this cross-sectional study could not identify causal relationships. Future research should investigate whether addressing PL can effectively increase physical activity levels.

## 5 Conclusion

This study revealed that the PL4L has a factor structure that is consistent with the concept of PL among Japanese adults. Additionally, PL correlates with the stages of change for participation in regular physical activity. These findings may help evaluate PL and clarify its role in promoting physical activity among adults.

## Data Availability

The raw data supporting the conclusions of this article will be made available by the authors, without undue reservation.

## Footnotes

^1^ http://www.myvoice.co.jp
