# Supplementary material for: Examining the factor structure of the Physical Literacy for Life self-assessment tool (PL4L) among Japanese adults and its relationship with the stages of change model for participation in regular physical activity
Source: Front Public Health. 2025 Mar 12;13:1505502. doi: 10.3389/fpubh.2025.1505502 (PMC11936968; doi:10.3389/fpubh.2025.1505502)
Supplement: Supplementary file 1 [file Data_Sheet_1.pdf]

Original	Japanese
**Physical Activity**（Number of days）<br>On how many of the last 7 days did you walk quickly, do sports or other physical activity for 30 minutes or longer?	**身体活動（日数）**<br>過去 7 日間のうち、早歩きなどの運動や各種スポーツのような身体活動を 30 分以上行った日数は何日ですか。
**Sedentary Behaviour**（Hours and minutes per day）<br>The following question is about the time you spent sitting on weekdays during the last 7 days. Include time spent at work, at home, while doing course work and during leisure time. This may include time spent sitting at a desk, visiting friends, reading, or sitting or lying down to watch television.<br>During the last 7 days, how much time did you usually spend sitting on a weekday?	**座位行動（1 日あたりの時間：〇時間〇分）**<br><br>過去 7 日間のうち、平日に座って過ごしていた時間は 1 日平均どのくらいありましたか。<br>※なお、職場や自宅で、何か作業をしていた時や趣味などを楽しんでいた時を含みます。<br>※また、机に向かって座っていた時、友人のもとを訪ねた時、あるいは読書やテレビを視聴するために座ったり横になったりしていた時なども含まれます。
**〈Physical Domain〉**<br>The Physical Learning domain is about being able to:<br>- apply various combinations of movement competencies that a situation or environment requires;<br>- manipulate the body and different objects across a variety of movement environments (land, water, air);<br>- acquire components of fitness to successfully participate in a range of physical activities.	**〈身体領域〉**<br>「身体領域」では、以下のことがどの程度できるかについてうかがいます。続く質問をお答えください。<br>- 状況や環境に応じ、さまざまな動作を組み合わせて運動することができるか。<br>- さまざまな環境（地上、水中、空中など）で自身の身体やいろいろな物（道具）を操作できるか。<br>- さまざまな身体活動に問題なく参加できる体力があるか。
**1) Strength**<br>Capacity of muscle(s) to exert force against an object	**1） 筋力**<br>あなたの筋力（筋肉が力を発揮する能力）について最もあてはまるレベルをお答えください。
**Level1 option**: I have difficulty using my strength in simple daily activities (e.g. can't carry shopping bags or do a sit up; can't keep correct posture while seated)	**レベル 1**: 日常生活の場面で、力を発揮することが難しい（例: 買い物袋を持ち運ぶことができない、起き上がることができない、座っている時に適切な姿勢を維持できないなど）
**Level2 option**: I can use my strength in general contexts of physical activity (e.g. push-ups, sit-ups, pull-ups, gardening/shoveling)	**レベル 2**: 通常の身体活動の場面で、力を発揮することができる（例: 日常的な家事作業や、腕立て伏せ、腹筋運動、懸垂運動など）
**Level3 option**: I'm able to use my strength in challenging contexts of physical activity (e.g. lifting heavy weights, rock climbing, circuit training, vigorous activity)	**レベル 3**: 激しい身体活動の場面で、より大きな力を発揮することができる（例: 重い物を持ち上げる、ウエイトトレーニングやサーキットトレーニングといった激しい身体活動など）

Original	Japanese
**2) Stamina**<br>Capacity to sustain prolonged physical effort	**2) スタミナ**<br>あなたのスタミナ(長時間にわたり身体活動を持続する能力)について最もあてはまるレベルをお答えください。
**Level1 option:** I have difficulty making light efforts (e.g. can't go up a flight of stairs without getting out of breath; have to take breaks while walking around)	**レベル 1:** 軽い身体活動でも継続することが難しい(例: 休憩をとりながら出ないと階段を登ったり歩き回ることができないなど)
**Level2 option:** I can participate in activities that take moderate physical effort and make me breathe somewhat heavier than normal (e.g. carrying light loads, running/bicycling at a regular pace)	**レベル 2:** 少し息がはずむような身体活動ができる(例: 軽い荷物を運ぶ、一定のペースで走る/自転車をこぐなど)
**Level3 option:** I can participate in diverse challenging activities that take hard physical effort and make me breathe much harder than normal (e.g. heavy lifting, digging, aerobics, fast running/bicycling for extended periods of time)	**レベル 3:** 身体的にきついと感じ、息がかなりはずむような身体活動ができる(例: 重い荷物を運ぶ、エアロビクス、長時間にわたり速く走る/自転車をこぐなど)
**3) Movement skills**<br>Movement skills that allow the person to move independently from one spot to another	**3) 自力での移動**<br>あなたのある地点から別の地点へ移動する能力について最もあてはまるレベルをお答えください。
**Level1 option:** I have difficulty moving (e.g need assistance walking)	**レベル 1:** 人の助けを借りずに移動することが難しい(例: 歩行のために補助は必要であるなど)
**Level2 option:** I can move using different skills in general contexts of physical activity (e.g. rolling, jumping, running, climbing)	**レベル 2:** 通常の身体活動場面で、さまざまな動作で移動できる(例: 走る、跳ぶ、登るなど)
**Level3 option:** I can move using different skills in challenging contexts of physical activity (e.g. running in a competitive game or race, climbing a difficult wall, sprinting to catch a bus)	**レベル 3:** 激しい身体活動の場面であっても、さまざまな動作で移動できる(例: 試合やレースで走る、バスに乗るために疾走する、登りにくい壁を登るなど)
**4) Movement using an object**<br>Movement skills used to move on, in or with equipment from one place to another	**4) 道具を使った移動**<br>あなたの用具を使用してある地点から別の地点へ移動する能力について最もあてはまるレベルをお答えください。
**Level1 option:** I have difficulty using objects/an apparatus to move from one place to another (e.g. can't ride a bicycle or skate)	**レベル 1:** 用具を使用して移動することが難しい(例: 自転車に乗ることができないなど)

Original	Japanese
**Level2 option:** I can use objects/an apparatus to move from one place to another in general contexts of physical activity (e.g. bicycle, skateboard, surf, ski, roller skates)	**レベル 2:** 通常の身体活動場面で、用具を使用して移動することができる(例: 自転車、キックボード、スケートボード、スキーなど)
**Level3 option:** I can use objects/an apparatus to move in challenging contexts of physical activity (e.g. cycling on a hilly trail, skiing on a difficult course, riding a horse)	**レベル 3:** 用具を使用した身体活動が難しい場面であっても、移動することができる(例: 坂道で自転車をこぐ、難しいコースでスキーをするなど)
**5) Coordination & balance**<br>Capacity to move two or more body parts in a controlled, smooth and efficient manner	**5) コーディネーションとバランス**<br>あなたのコーディネーション(調整)能力とバランス(平衡)能力について最もあてはまるもレベルをお答えください。
**Level1 option:** I have difficulty coordinating my movements and balancing myself (e.g. can't hold myself upright without assistance)	**レベル 1:**自分の動きを調整したりバランスをとったりすることが難しい(例: 補助なしで立っていることができない)
**Level2 option:** I can coordinate my movements and balance myself in general contexts of physical activity (e.g. dance, batting a ball, walking on a beam, gymnastics)	**レベル 2:** 通常の身体活動場面で、自分の動きを調整したりバランスをとったりすることができる(例: ダンスをする、平均台の上を歩く、体操をするなど)
**Level3 option:** I can coordinate my movements and balance myself in challenging contexts of physical activity (e.g. dancing a fast choreography, balance on a moving platform)	**レベル 3:** 難易度の高い身体活動場面であっても、自分の動きを調整したりバランスをとることができる(例: スピードのある振り付けでダンスをする、動く台の上でバランスをとる)
**6) Object manipulation skills**<br>Movement skills that use a body part(s) to move or manipulate an object	**6) 操作能力**<br>あなたの物を操作する能力について最もあてはまるもレベルをお答えください。
**Level1 option:** I have difficulty manipulating objects in physical activities (e.g. can't throw or catch an object)	**レベル 1:** 身体活動において、物を操作することが難しい(例: 物を投げられない、キャッチできない)
**Level2 option:** I can use/manipulate objects in general contexts of physical activities (e.g. throw/catch/dribble/kick a ball, twirl a baton, bat a ball)	**レベル 2:** 通常の身体活動場面で、物を操作することができる(例: ボールを投げる/キャッチする/ドリブルする/キックする、バトンを回す、ボールを打つ)
**Level3 option:** I can manipulate objects in challenging contexts of physical activity (e.g. control a ball in a competitive game, use an object in a choreography)	**レベル 3:** 難易度の高い身体活動場面であっても、物を操作することができる(例: 試合でボールをコントロールする、物を操作しながら踊る)

Original	Japanese
**〈Emotional Domain〉**<br>The Emotional Learning domain is about being able to:<br>- acquire satisfaction and enjoyment from different movement experiences<br>- understand emotional responses, such as empathy and sensitivity, during movement and physical activities<br>- manage physical responses, such as fatigue or pain, during movement and physical activities<br>- persist with movement in the face of difficulty, challenge or failure, in the belief that improvement will come with persistent learning and effort	**〈感情領域〉**<br>「感情領域」では、以下のことについてうかがいます。続く質問をお答えください。<br>- さまざまな運動経験から満足感と楽しみを得ることができるか。<br>- 身体活動を行っている際に、共感や感受性などの、感情を理解できるか<br>- 身体活動を行っている際に、疲労または痛みなどの身体的な反応に対応できるか。<br>- 困難、難題、または失敗に直面しても努力して学習し続けることによって向上すると信じて、身体活動を続けられるか。
**1) Motivation**<br>Reasons for engaging in movement and physical activity	**1) モチベーション**<br>あなたが身体活動を行う理由について最もあてはまるレベルをお答えください。
**Level1 option:** I don't feel like participating in physical activities/movement	**レベル 1:** 身体活動を行う気にならない
**Level2 option:** I participate in physical activity because it brings me approval, recognition or rewards from others	**レベル 2:** 身体活動を行うのは、他者からの承認、評価、報酬が得られるからである
**Level3 option:** I participate in physical activity because it brings me joy, pleasure and self-realization	**レベル 3:** 身体活動を行うのは、喜び、楽しみ、自己実現が得られるからである
**2) Confidence (self-efficacy)**<br>A belief in your capacity to perform in movement and physical activity setting/s	**2) 自信**<br>あなたの身体活動場面における(自分の能力を発揮できる)自信について最もあてはまるレベルをお答えください。
**Level1 option:** I'm not confident I can participate in physical activities	**レベル 1:** 身体活動を行う自信がない
**Level2 option:** I am confident I can participate in general contexts of physical activity (e.g. housekeeping activities, active commuting, sports participation, fitness activities)	**レベル 2:** 通常の身体活動場面(例: 自転車や徒歩による通勤・通学、スポーツ、フィットネスなど)であれば、自分ならできるという自信がある
**Level3 option:** I'm confident I can participate in physical activities in challenging situations (e.g. new or unknown contexts, lack of time, feeling tired)	**レベル 3:** 身体活動を行うことが難しい場面(例: 新しい、未知の状況、時間がない、疲れているなど)であっても、自分ならできるという自信がある

Original	Japanese
**3) Physical regulation**<br>Perceiving, adjusting and controlling internal physical signals, such as fatigue and pain, in movement and physical activity contexts During Physical Activities:	**3）身体的な調整・制御**<br>あなたの身体活動場面における身体の変化（疲労や痛みなど）への気づきおよびその調整・制御について最もあてはまるレベルをお答えください。
**Level1 option:** I have difficulty recognizing when I'm fatigued	**レベル 1:** 身体活動場面において自分が疲れていることに気づくのが苦手である。
**Level2 option:** I can recognize when I'm fatigued	**レベル 2:** 身体活動場面において自分が疲れていることがわかる。
**Level3 option:** I can manage my fatigue using different strategies (e.g. pacing myself, controlling my breathing)	**レベル 3:** 身体活動場面において様々な方法（例： ペース配分、呼吸のコントロールなど）で自分の疲労を管理できる
**4) Emotional regulation**<br>Capacity to adjust and control emotions (e.g. happiness, sadness, fear, anger) and resulting behaviors in relation to movement and physical activity contexts During Physical Activities:	**4）感情的な調節・制御**<br>あなたの身体活動に関連する感情（例： 喜び、悲しみ、恐れ、怒りなど）とその結果としての行動の調整・制御について最もあてはまるレベルをお答えください。
**Level1 option:** I have difficulty recognizing my emotions	**レベル 1:** 身体活動場面において自分の感情の変化に気づくのが苦手である
**Level2 option:** I can recognize my emotions	**レベル 2:** 身体活動場面において自分の感情の変化を認識できる
**Level3 option:** I can manage my emotions using different strategies (e.g. focusing on positive thoughts, calming down before reacting)	**レベル 3:** 身体活動場面において自分の感情をコントロールすることができる（例： ポジティブな考え方に意識を向ける、感情のまま行動する前に心を落ち着かせる）
**〈Cognitive Domain〉**<br>The Cognitive Learning domain is about being able to:<br>- Reason and make appropriate decisions, knowing how, when, and why to perform certain movement skills<br>- Adhere to rules and to apply tactics within a game<br>- Set and implement goals within Physical Activities<br>- Use knowledge regarding Physical Activities	**〈認知領域〉**<br>「認知領域」では、以下のことについてうかがいます。続く質問にお答えください。<br>- どのように、いつ、なんのために、どのような動きをするのかを知り、適切な判断を下すことができるか。<br>- ルールを守り、ゲームの中で戦術を利用できるか。<br>- 身体活動の中で目標を設定し、実行できるか。<br>- 身体活動に関する知識を活用できるか。
**1) Knowledge**<br>Factual knowledge and information that a person knows and can convey about physical activities (e.g. knowing that benefits of physical activity include physical, emotional, social and cognitive benefits)	**1）知識**<br>あなたが身体活動に関する知識や情報（例： 身体的、感情的、社会的、認知的なメリットなど）を知っているか、また、伝えることができるかについて最もあてはまるレベルをお答えください。

Original	Japanese
**Level1 option:** I have difficulty recognizing benefits of physical activity	**レベル 1:** 身体活動のメリット（効果）を理解していない
**Level2 option:** I know the general benefits of physical activity	**レベル 2:** 身体活動の一般的なメリット（効果）を理解している
**Level3 option:** I can relate different types of physical activities with their specific benefits (e.g. sports, rhythmic activities, active commuting)	**レベル 3:** さまざまな種類の身体活動（例： 自転車や徒歩による通勤・通学、リズミカルに身体を動かす、各種スポーツなど）とそれぞれのメリット（効果）を関連づけることができる
**2) Rules (& tactics)**<br>Regulations, guidelines or principles governing conduct or procedure within activities. May be explicit, spoken or unspoken (e.g. safety and hygiene rules, rules in games). Planned and ad hoc decisions and actions, employed in the moment for the pursuit of goals	**2） ルールと戦術**<br>あなたが身体活動の場面で遵守すべき規則、ガイドライン、原則、あるいは手続き（例： 安全や衛生面のルール、試合中のルールなど、それらは、明示的なもの、口頭で言われるもの、あるいは暗黙の了解の場合がある）、また目標達成のために、その場で行われる計画的かつ臨機応変な意思決定や行動について最もあてはまるレベルをお答えください。
**Level1 option:** I have difficulty recognizing the rules or tactics of the physical activities I participate in	**レベル 1:** 身体活動に参加するためのルールや戦術を認識することが苦手である
**Level2 option:** I know and adhere to the rules and tactics of the physical activities I participate in	**レベル 2:** 行っている身体活動のルールや戦術を理解し守っている
**Level3 option:** I can adjust rules and tactics to the specific physical activity contexts I'm in	**レベル 3:** 自分が行う身体活動の場面や状況に合わせて、ルールや戦術を調整できる
**3) Strategy**<br>Capacity to set and implement goals within activities (e.g. planning route to school/work in active commuting, developing an exercise plan, winning a game)	**3） 戦略**<br>あなたが身体活動において目標を設定して実行する能力（例： 自転車や徒歩による通勤・通学のルートを計画する、エクササイズの計画を立てる、試合に勝つ）について最もあてはまるレベルをお答えください。
**Level1 option:** I have difficulty planning or reaching my goals in physical activities	**レベル 1:** 身体活動において、計画を立てて自分の目標を達成することが苦手である
**Level2 option:** I know how to plan and reach my goals in physical activities	**レベル 2:** 身体活動において、計画を立てて自分の目標を達成する方法を知っている
**Level3 option:** I can adapt my plans to unknown circumstances (e.g. adapt a training plan to train at home, change a practice location according to weather conditions)	**レベル 3:** 状況にあわせて自分の計画を柔軟に変えることができる（例： 自宅でトレーニングするためにトレーニング計画を柔軟に変える、天候に応じて練習場所を変えるなど）

Original	Japanese
**〈Social Domain〉**<br>The Social Learning domain is about being able to:<br>- utilize the natural and built environment for connection to others and place through movement and physical activity<br>- lead others in collaborative, ethical and inclusive behaviors in physical activity, including understanding when to be a team member or a leader<br>- share and learn from experiences from your own and other cultures<br>- exhibit fair play and ethical behaviour in a variety of physical activities and environments.	**〈社会領域〉**<br>「社会領域」では、以下のことについてうかがいます。続く質問をお答えください。<br>- 身体活動を用いて他の人と関わり合えるように自然環境を利用したり、環境を整えることができているか。<br>- チームメンバーとして、またはリーダーとしての役割を理解することを含め、身体活動において協調的、倫理的、インクルーシブ（共に支え合う）な行動で他者を先導できているか。<br>- 自国や他国の文化から得た経験を共有し、そこから学ぶことができているか。<br>- さまざまな身体活動や環境において、フェアプレーと倫理的な行動を示すことができているか。
**1) Ethics**<br>Moral principles that govern a person's behaviour relating to fairness and justice	**1）倫理**<br>あなたの公平さや正義に関わる道徳的原理について最もあてはまるレベルをお答えください。
**Level1 option:** I have difficulty recognizing principles of fairness, respect and inclusion in physical activities	**レベル 1:** 身体活動において、公正であり、他者を尊重し、インクルーシブ（共に支え合う）であることが苦手である
**Level2 option:** I generally apply principles of fairness and inclusion in physical activities	**レベル 2:** 身体活動において、公正であり、インクルーシブであろうとしている
**Level3 option:** I can use strategies to improve conditions for respect, fairness and inclusion in physical activities	**レベル 3:** 身体活動において、他者の尊重、公正、インクルーシブの状況を改善に向かわせることができる
**2) Society & culture**<br>Appreciation of cultural values that exist within groups, organizations and communities (e.g. symbols, chants, traditional dances and games, salutations, habits)	**2）社会と文化**<br>あなたの文化的な価値観（シンボル、ゲーム、礼儀作法、習慣など）の正しい理解について最もあてはまるレベルをお答えください。
**Level1 option:** I have difficulty recognizing cultural values and practices in physical activities	**レベル 1:** 身体活動における文化的な価値観や習慣を認めることが難しい
**Level2 option:** I can recognize and participate in cultural values and practices in physical activities	**レベル 2:** 身体活動における文化的な価値観や習慣を認識し、それに参加することができる
**Level3 option:** I respect and encourage diversity of cultural values and practices in physical activities	**レベル 3:** 身体活動における文化的な価値観や習慣を尊重して積極的に取り組む姿勢でいる

Original	Japanese
**3) Collaboration**<br>Social skills for successful interaction with others, including communication, cooperation, leadership and conflict resolution	3) **協力**<br>あなたの他者と上手に交流する社会的スキル（コミュニケーション、協力、リーダーシップ、争いごとの解決など）について最もあてはまるレベルをお答えください。
**Level1 option:** I have difficulty collaborating with others during physical activities	**レベル 1:** 身体活動において、他者と協力・協調することが苦手である
**Level2 option:** I respect and collaborate with others during physical activities (e.g. respect another participant's space, share equipment or ball)	**レベル 2:** 身体活動において、他者を尊重して協力・協調できる（例： 他の参加者のスペースを尊重する、道具やボールを共有する）
**Level3 option:** I actively support and encourage the success of others during physical activities (e.g. help another participant)	**レベル 3:** 身体活動において、他者がうまくいくように積極的にサポートし、励ますことができる
