# Supplementary material for: Examining the factor structure of the Physical Literacy for Life self-assessment tool (PL4L) among Japanese adults and its relationship with the stages of change model for participation in regular physical activity
Source: Front Public Health. 2025 Mar 12;13:1505502. doi: 10.3389/fpubh.2025.1505502 (PMC11936968; doi:10.3389/fpubh.2025.1505502)
Supplement: Supplementary file 2 [file Data_Sheet_2.pdf]

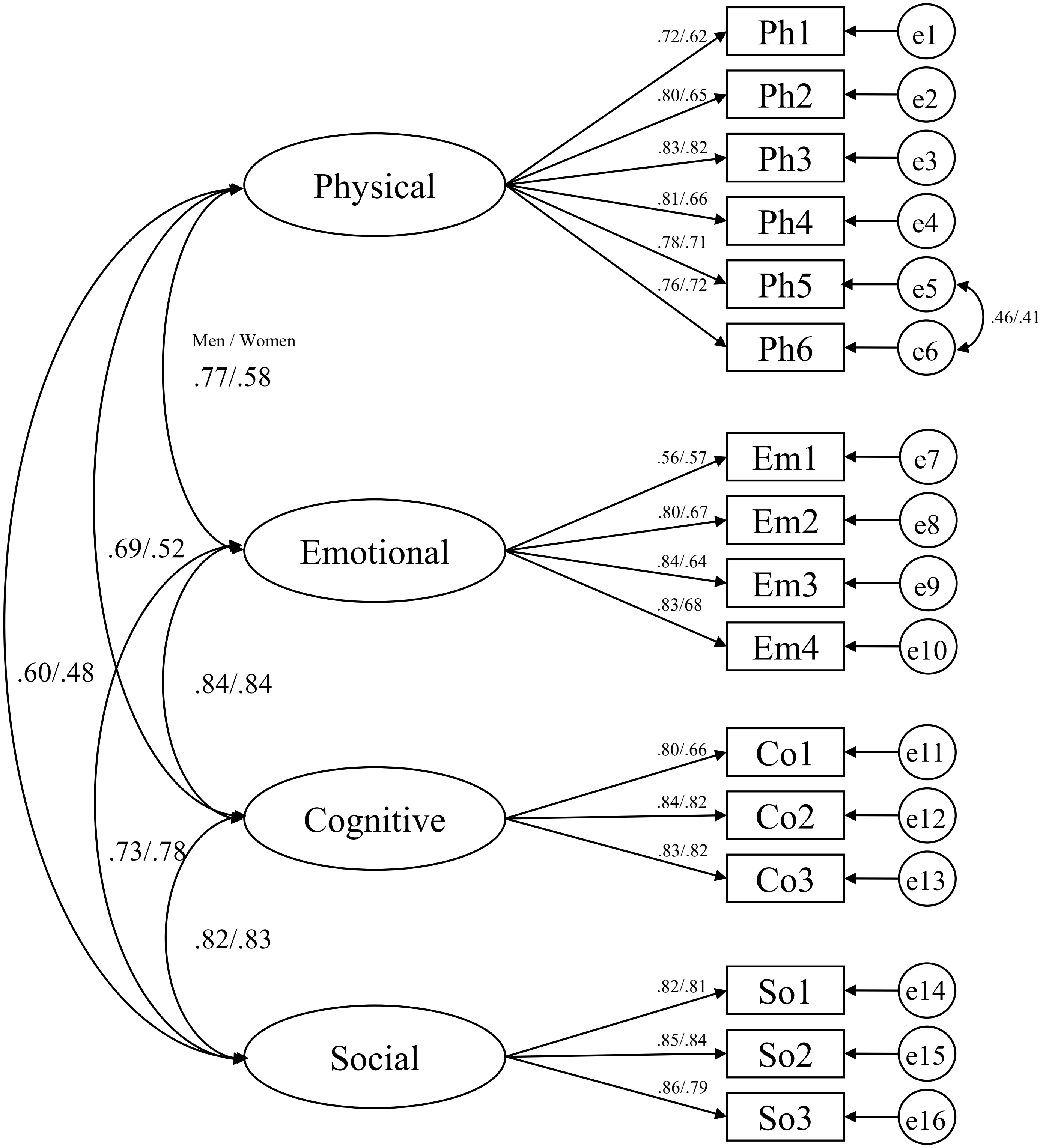

Men GFI = 0.937 AGFI=0.912 CFI = 0.970 RMSEA = 0.058
Women GFI = 0.944 AGFI=0.921 CFI = 0.962 RMSEA = 0.053

Supplementary2： Final factor model of the Physical Literacy for Life self-assessment tool among Japanese adults (stratified analysis by sex)

Figure legend: Goodness-of-fit indices (GFI, AGFI, CFI) suggest better model fit with higher values, whereas RMSEA indicates better fit with lower values. Factor loadings from latent variables to observed variables are meaningful when approximately 0.40 or greater, and all paths are statistically significant ($p < 0.001$)
